# Specific expression pattern of IMP metabolism related-genes in chicken muscle between cage and free range conditions

**DOI:** 10.1371/journal.pone.0201736

**Published:** 2018-08-22

**Authors:** Tao Zhang, Hongzhao Lu, Ling Wang, Meichen Yin, Likai Yang

**Affiliations:** School of Bioscience and Engineering, Shaanxi University of Technology, Hanzhong Shaanxi, People's Republic of China; Qingdao Agricultural University, CHINA

## Abstract

Inosine monophosphate (IMP) is a key factor affecting the fleshy flavor of meat; meanwhile, the free-range mode is an efficient strategy to improve muscular IMP content. To assess expression differences in IMP metabolism-related genes under different feeding patterns, Illumina Nextseq 500 sequencing was used to catalog the global gene expression profiles of muscle samples from Lueyang black-bone chicken under free-range and caging conditions. A total of 15510 unigenes were assembled, with 13423 (86.54%) and 6088 (39.25%) unigenes correctly annotated in the GO and KOG databases, respectively. Next, the "purine metabolism" pathway in the "nucleotide metabolism group" was assessed in depth. Through Kyoto Encyclopedia of Genes and Genomes (KEGG) analysis, we retrieved 172 nucleotide- and 5 purine- metabolism related genes that were differentially expressed in muscle samples from free-range and caged chickens. At 60-day-old, AMPD1, NT5C1A and ENTPD8 showed higher levels in the free-range group, while only ENTPD8 was upregulated in 120-day-old chickens. In addition, GART, GARS and ADSL in free-range chickens showed higher levels compared with caged animals. Furthermore, IMPDH levels in free-range chicken were lower than those of caged chicken. Real-time quantitative polymerase chain reaction (qPCR) was used to validate the above findings. These results revealed a set of differentially expressed genes potentially related to IMP metabolism in chicken under different breeding modes, providing novel insights into controlling IMP levels in chicken meat.

## Introduction

In the past few decades, scientists have mainly focused on the appearance, growth performance and feed reward of broiler chickens, and the growth rate and muscle production yields of chicken have been greatly improved. However, it is unfortunate that the quality of chicken meat decreases significantly with increasing growth rate. Therefore, it remains challenging to improve chicken meat quality while maintaining a high growth rate. Currently, wholesalers have acknowledged delicate flavor as an important indicator of meat quality [1]. Studies demonstrated that inosine monophosphate (IMP) confers the distinct flavor in the cooking process. Therefore, IMP content is an important index for meat quality assessment. IMP synthesis is a very complex process regulated by various factors in the muscle [2]. IMP is degraded to hypoxanthine by a series of enzymes. In livestock and poultry, IMP content is affected by strain, sex, feeding methods and storage conditions after slaughter [3,4]. It is well-known that meat quality is different in domestic animal and poultry breeds, as assessed by tenderness, IMP content, water holding capacity (WHC) and abdominal fat, among other indexes [5,6]. Studies reported that free-range chicken are superior to caged counter parts in tender and delicious meat, because of lower subcutaneous and abdominal fat levels [7]. In addition, feeding and management can also significantly affect IMP synthesis and degradation in the muscle. In poultry production, caging and free-range methods significantly affect skeletal muscle development, and indirectly influence muscular IMP metabolism. It is known that the feeding mode is a key factor affecting meat quality, and people prefer to select free-range animal-sourced food [8]. Lueyang black-bone chicken is a famous native chicken breed, which is found in Lueyang county of Hanzhong city in the Shaanxi province of China. This chicken breed has excellent meat quality, and is well-known for its characteristic eight black parts, including skin, beak, thighs, claws, tongue, cockscomb, feathers and bones. Previous studies have shown that IMP content is higher in this breed compared with Taihe Silky fowl and Chuannan black-bone chicken. To assess differences in IMP content among distinct animal breeds and feeding modes, multiple studies have focused on gene polymorphisms of IMP metabolic related enzymes, such as GARS, AIRS, GART and GPAT, and the expression patterns of these genes [9,10]. However, the key regulatory pathways that affect IMP metabolism have not been fully defined, especially in Lueyang black-bone chicken. In this study, caging and free-range modes were designed for Lueyang black-bone chicken in a relatively stable external environment, and IMP levels were assessed in skeletal muscles at different ages under the two feeding modes. To assess the effects of feeding methods on the quality of chicken meat, key regulators of IMP synthesis, degradation and utilization during muscle development were examined in Lueyang black-bone chicken at 60 and 120 days, respectively, by RNA-seq. Our findings reveal candidate genes, and provide novel and useful insights into IMP metabolic regulation and chicken breeding.

## Material and methods

### Ethics statement

All animal experiments were approved by the Institutional Animal Care and Use Committee of Shaanxi University of Technology, and performed according to the National Institutes of Health guidelines for animal experiments.

### Sample collection and total RNA extraction

All Lueyang black-bone chicken samples were from Shaanxi Tonghui Agricultural Co, Ltd (Lueyang County, Hanzhong City, Shaanxi Province, China). The experiment divided into four groups: 60 days old caged group, 120 days old caged group, 60 days old free-range group, 120 days old free-range group. Totally 12 cocks were assigned randomly into these four groups, with 3 cocks in each group. Caged chickens were kept in a traditional cage indoor, and the raising density of each cage was about 8 birds/m2. Free-range chickens were maintained in a similar indoor house at night with the same density, but they also had a free-range area during day time. The free area was mainly low shrub land with small weeds, little stones, sands, and the raising density was about 1 bird/m2. 5g of tissue samples of breast and thigh muscles were collected from four groups, respectively. A part of each sample was immediately snap-frozen in liquid nitrogen for cDNA library preparation, and the other part was used to assess IMP content by HPLC.

Breast and thigh muscle samples were homogenized, and total RNA from different tissues was extracted with TRIzol reagent (Takara, Dalian, China) according to the manufacturer’s instructions. Then, RNA quality and amounts were assessed by gel electrophoresis and on a Nano Drop 2100 (Thermo, Madison, USA), respectively.

### cDNA library preparation and sequencing

Total RNA samples were treated with DNase, and mRNA was purified with oligo dT magnetic beads. The isolated mRNA was fragmented into about 375bp by chemical reagents at high temperature. Double-strand cDNAs were synthesized with random hexamer primers by PCR. The 3′ terminus of purified cDNA fragments were repaired and added an A-tail, followed by ligation to sequencing adaptors with T and labeling. The fragments with adaptors were enriched and amplified by PCR, resulting in the RNA-seq libraries of Lueyang black-bone chicken muscle. Quantification of these libraries was performed on a QuantiFluor-ST fluorometer (Promega, E6090) with Quant-It Pico Green dsDNA Assay Kit (Invitrogen, P7589). The quality of the libraries was assessed on an Agilent 2100 Bioanalyzer (Agilent Technologies, Santa Clara, CA, USA).

### IMP content analysis by HPLC

Muscle samples (5 g) were placed in a clean container, mashed, and added 20 mL of 6% perchloric acid. The homogenized samples were centrifuged at 4000 r/min for 15 min and the supernatant collected. The resulting precipitate was treated again to obtain another supernatant, which was combined with the previous to form the sample extract. The total extract was centrifuged again, and the resulting supernatant was collected. The pH of the extract was adjusted to 6.5. Standard solutions and the sample extract were analyzed by high performance liquid chromatography (HPLC) according to standard protocols [11].

### Sequence annotation and identification of differentially expressed genes

Library sequencing was performed on an Illumina Nextseq 500 (Personal bio, Shanghai, China), and raw sequencing data were stored in the FASTQ format. These data included the ID, sequence and quality of each base. The raw data were cleaned by removing adaptors and low quality reads, and analyzed with FastQC (http://www.bioinformatics.babraham.ac.uk/projects/fastqc/). All clean reads were mapped to the chicken genome assembly Gallus_gallus. Galgal4 (downloaded fromEnsembl:ftp.ensembl.org/pub/release-82/fasta/gallus_gallus/dna/) using bowtie2/tophat2, followed by assembly and evaluation [12]. The fragments per kilobase of exon per million fragments mapped (FPKM) threshold for gene detection was set at >0.5[13]. Differentially expressed genes (DEGs) were identified with the DESeq software (http://www-huber.embl.de/users/anders/DESeq/). To identify transcripts with differential expression among the four samples, the FDR was used to adjust the p value threshold in multiple hypothesis testing. Genes with a FDR adjusted P-value (P<0.05) and fold change ≥2 were considered to show significant differences [14].

### Functional analysis of unigenes

All assembled unigenes were identified with the BLASTX program in the GO and KEGG pathways, with a cut-off E-value of 10^−5^[15,16]. All ontology functions were confirmed by a combination of results obtained in the NCBI database. Functional categorization by gene ontology (GO) terms was carried out according to molecular function, biological process and cellular component [17]. To assess the biological functions and signal pathways of related genes in Lueyang black-bone chicken, all differentially expressed genes were subjected to screening and examined in the KEGG database [18].

### Quantitative-PCR for candidate genes

According to results of transcriptome analysis, seven candidate genes involved in inosinic acid metabolism related KEGG pathways were screened. The expression levels of these candidate genes were assessed by qRT-PCR in different samples. Real-time quantitative PCR was performed in triplicate with a SYBR green kit (Invitrogen, Thermo Fisher) on an ABI StepOne plus system, with chicken β-actin selected as the reference gene. The 20μL PCR reactions included 10μL of SYBR Premix ExTaq II (TaKaRa, Dalian, China), 3.2μL (10 pmol/μL) of each specific forward and reverse primers, 2μL (10 ng/μL) of diluted cDNA and 4.8μL of RNase free water. Cycling conditions were 95°C for 5 min, followed by 40 cycles of 95°C for 10 sec, 58°C for 20 sec and 72°C for 15 sec. The 2^-ΔΔCT^ method was employed to estimate the relative expression levels of various genes.

## Results

### Chicken body weight and muscular inosinic acid content

First, we compared live body weights (BWs) of Lueyang black-bone chickens at the ages of 60 and 120 days, respectively, between the two feeding modes. The results showed that live BWs in the caging group were much higher than those of free-range animals (Table 1). In order to evaluate the flavor quality of chicken meat under different feeding patterns, IMP contents in breast and thigh muscle samples from 60- and 120-day-old animals were detected by HPLC, respectively. The data showed that IMP content in the breast muscle increased with age in both feeding patterns. Surprisingly, IMP content in the thigh muscle showed an opposite pattern with age between both feeding patterns. While IMP content in the thigh muscle increased with age in free-range animals (921.71±100.18), it decreased with age in caged counterparts (364.15±48.60). Comparing the two feeding modes, there were no significant differences(p>0.05) in IMP contents in breast and thigh muscles between the two breeding methods at 60 days. However, IMP levels were significantly different (p<0.01)in breast and thigh muscles between the two feeding modes at 120 days of age, with IMP contents in thigh muscle samples from the free-range group being significantly higher than those of caged animals (p<0.01) (Table 2).

**Table 1:** Live body weights in free-range and caged chickens.

Breeding Mode	Free-range		Caged	
	Ly60 (n = 3)	Ly120(n = 3)	Ly60 (n = 3)	Ly120(n = 3)
BW(g)	640.6±24.3	899.4±26.5	1012.6±43.2**	2102.8±56.9**

Note: BW, live body weight; Ly60, 60-day-old Lueyang black-bone chickens; Ly120, 120-day-old Lueyang black-bone chickens; Means differed significantly between the breeding methods at different ages (*p<0.05,**p<0.01).

**Table 2:** Determination of IMP content in the muscle.

Day-old	Caged		Free-range	
	Thigh muscle	breast muscle	Thigh muscle	breast muscle
60d	648.26±85.36	523.68±90.52	609.42±34.25	552.00±67.23
120d	364.15±48.60^aa^^,^ ^bb^	894.46±56.18^aa^^,^^bb^	921.71±100.18^aa^^,^^bb^	1481.76±79.88^aa^^,^^bb^

Note

^a^p<0.05 and

^aa^p<0.01, comparing 60 and 120 days

^b^p<0.05 and

^bb^p<0.01, thigh and breast muscles for the caged and free-range groups at different ages were compared.

### Illumina sequencing and de novo assembly

Due to significant differences in IMP contents of chicken muscles between the two feeding patterns, the Illumina NexSeq 500 system was used to perform transcriptome analysis and identify differentially expressed transcripts of inosinic acid metabolism related genes in the muscle of Lueyang black-bone chicken under the two feeding modes. In this study, 34,316,006 and 39,610,822 raw reads were obtained for the cage group at 60- and 120-days, respectively, while 27,806,566 and 30,617,310 raw reads were found in the free-range group at 60- and 120-days, respectively. After removal of low-quality reads and those containing adaptors or N (bases read not certain), the libraries yielded 34,045,928, 39,163,520, 27,577,958 and 30,365,032 clean reads, respectively. Q30 values (reflecting a base recognition accuracy of 99.9%) were 92.64%, 91.79%, 92.34% and 92.37%, respectively (Table 3). After clustering by TGICL (Mortazavi et al., 2008), a total of 15,510 unigenes with an average length of 28942.31 were obtained, for a total length of 448, 837, 326 (Table 3).

**Table 3:** Muscle transcriptome properties.

Description	Caged		Free-range		Both
	Ly60	Ly120	Ly60	Ly120	
RIN(RNA integrity Number)	10.0	10.0	10.0	10.0	
Raw reads	34,316,006	39,610,822	27,806,566	30,617,310	
Clean reads	34,045,928	39,163,520	27,577,958	30,365,032	
Q30(Q-score)	92.64%	91.79%	92.34%	92.37%	
Total number of unigenes					15510
Total length of unigene					448,837,326
Max length of unigene					802394
Min length of unigene					141
Average length of unigene					28942.31

**Fig 1:**
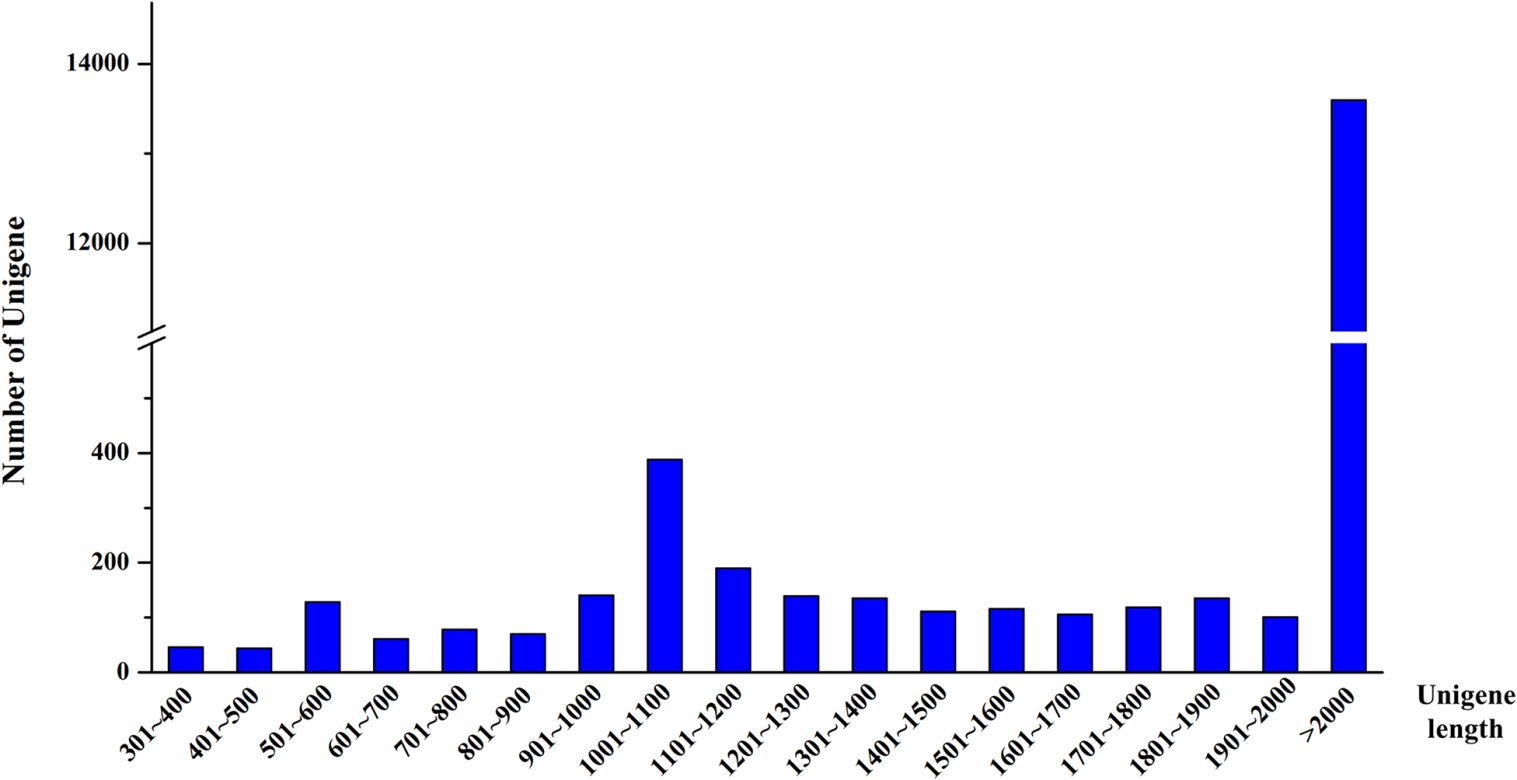
Histogram of unigene length distribution. The x-axis indicates the length range of unigenes; the y-axis denotes the number of unigenes.

Most of the assembled unigenes were more than 300 bp in size, with maximum and minimum lengths of 802,394bp and 141bp, respectively. Among these unigenes, 568 (3.66%) were 300 to 1000 bp long, 1540 (9.94%) were 1000 to 2000 bp long and 13,402 (86.40%) were longer than 2000 bp (Fig 1). These data indicated that the integrity of unigenes was relatively good.

Transcript abundance reflects gene expression levels; the higher the transcript abundance, the higher the level of gene expression. We compared the reads obtained by sequencing in current study, and found that transcript abundances in the four groups were all much higher, i.e. 71.08% and 70.16% in 60-and 120-day-old free-range chickens, and 70.37% and 68.04% in 60-and 120-day-old caged animals, respectively.

### Assembly evaluation and annotation

**Fig 2:**
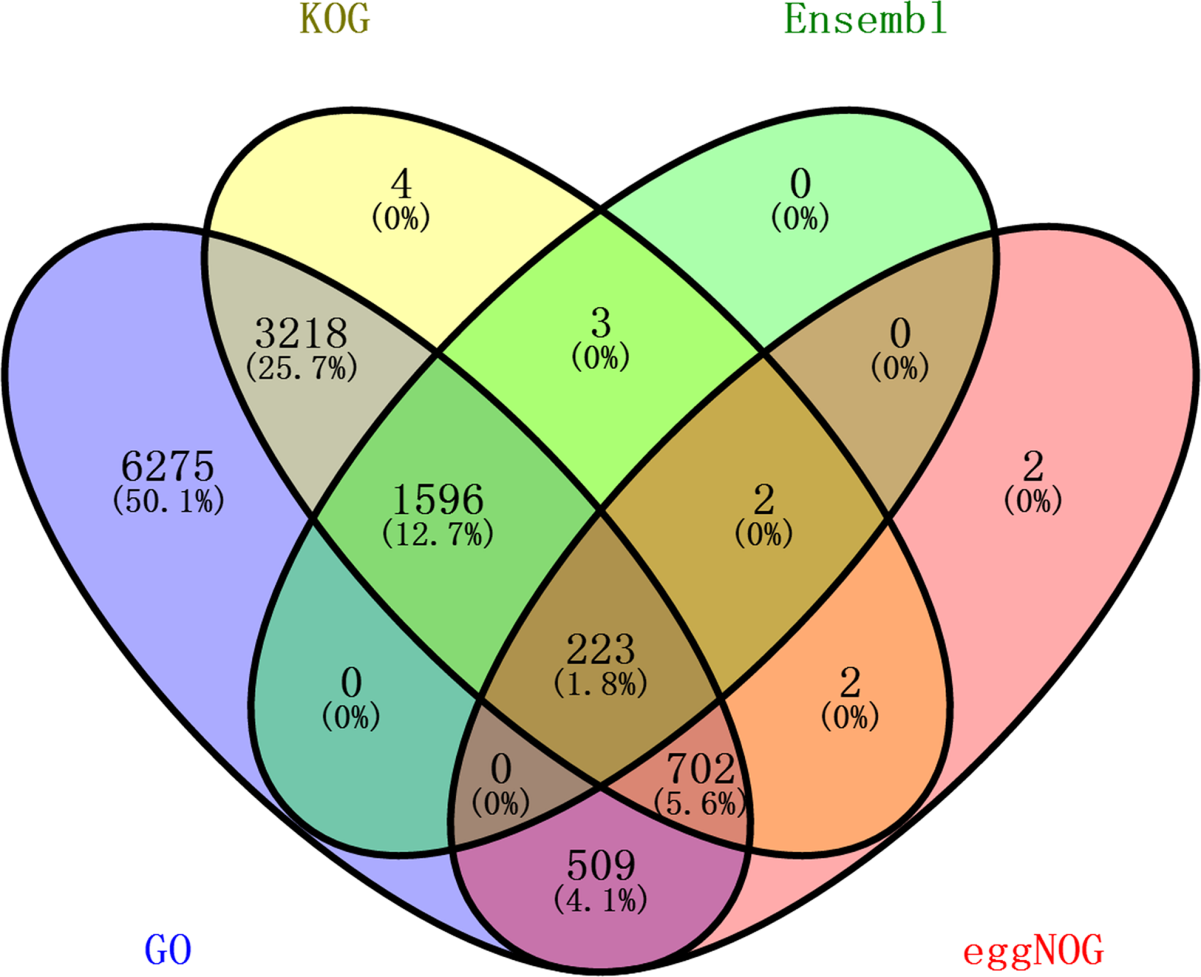
Comparison of the numbers of unigene annotations obtained from different databases. Every section displays the number of unigenes shared among the GO, KOG, Ensembl and EggNOG databases.

Based on transcriptome sequencing of the skeletal muscle, 15,510 annotated unigenes were obtained, and 86.54%, 39.25%, 12.66% and 9.57% received significant hits in the gene ontology (GO), clusters of orthologous groups for eukaryotic complete genomes (KOG), Ensembl, and evolutionary genealogy of genes: Non-supervised Orthologous Groups (EggNOG) databases, respectively (Table 4). Using the online Venny software, Venn diagrams were generated to visualize the number of unique or shared unigenes in the database (Fig 2). Out of 15,510 unigenes, only 283 had reference sequence in all four databases. However, most genes were correctly annotated in the GO and KOG databases, including 13423 (86.54%) and 6088 (39.25%) correctly annotated in the GO and KOG databases, respectively. These results indicated that at least 86.62% of the unigenes matched poultry, therefore supporting the validity of the current transcriptome data. Meanwhile, 2087 (13.45%) unigenes could not be annotated, maybe because they were too short to generate sequence matches, or represent new genes (Table 4).

**Table 4:** Annotation of unigenes BLAST against four different databases.

Database	Number of Annotated Unigenes	Annotated Unigene Ratio(%)
GO	13423	86.54%
KOG	6088	39.25%
Ensembl	1964	12.66%
EggNOG	1485	9.57%
unknown	2087	13.45%

### Gene function annotation

#### GO analysis

**Fig 3:**
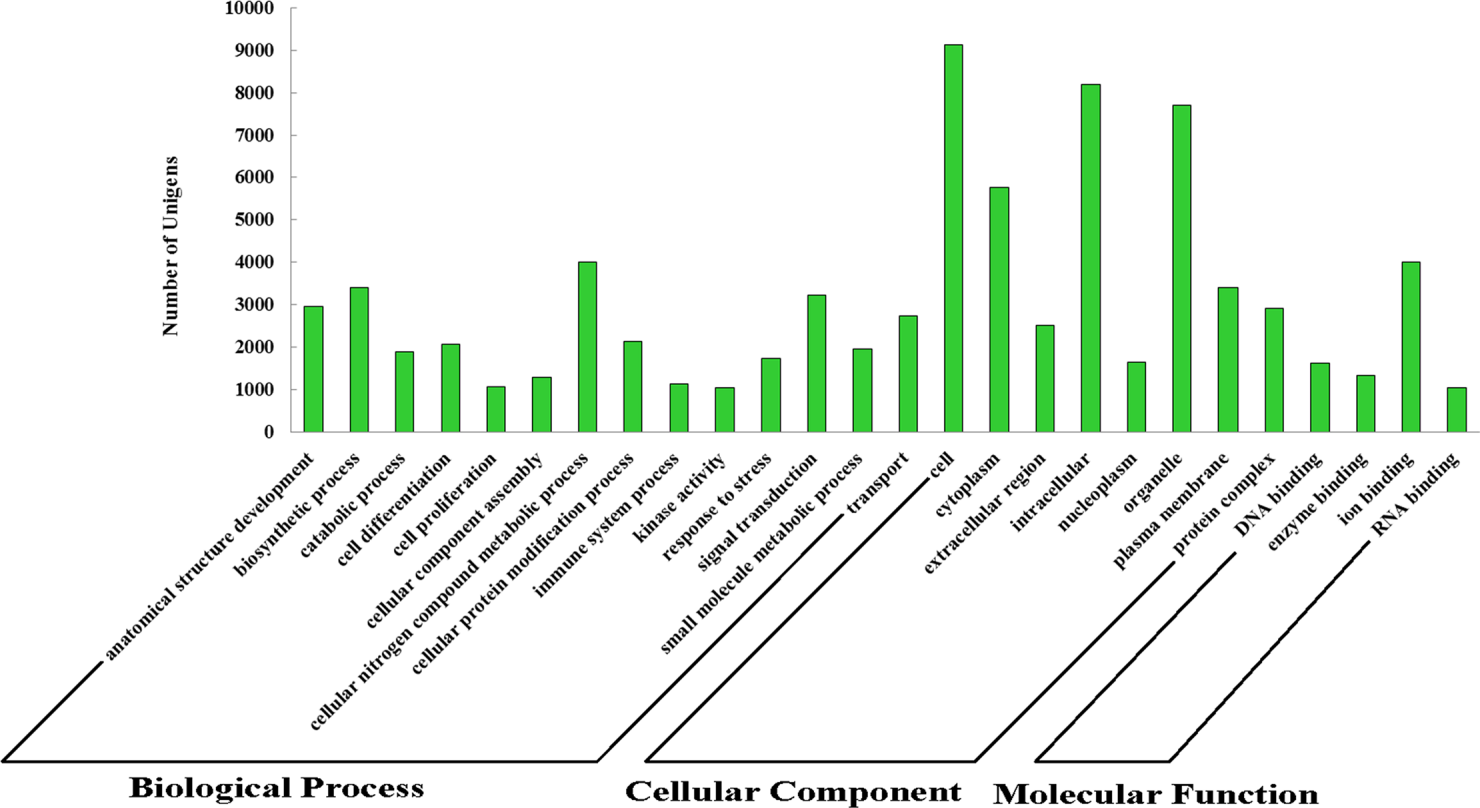
Gene Ontology classification. Unigenes were assigned to three categories, including biological processes, cellular components and molecular functions.

GO stands for Gene Ontology Consortium, an international standardized gene function classification system. We analyzed the unigenes obtained from the skeletal muscle transcriptome in the GO Slim database with the Blast2GO software [17], and detected 13423 unigenes. To achieve more annotation and classification according to GO functions, the 13423 unigenes were divided into 102 subcategories comprising three main categories, including biological processes, cell components and molecular functions (Fig 3). Meanwhile, one unigene was categorized into an additional GO term.

By statistically analyzing the number of genes in each category, the biological process category possessed the majority of genes (9328), with 4003 unigenes involved in the metabolic process of cellular nitrogen compounds. Further, the results showed that 1944 and 3397 unigenes belonged to the “small molecular metabolic process” and biosynthetic process” categories, respectively. In the category of cellular components, most genes were involved in “cell” (9132 unigenes), “organelle” (7701 unigenes) and “cytoplasm” (5752 unigenes) (Fig 3). In the category of molecular functions, the most common term was " ion binding", into which 3984 unigenes were classified, followed by the term “enzyme binding” (871 unigenes)

#### KO analysis

**Fig 4:**
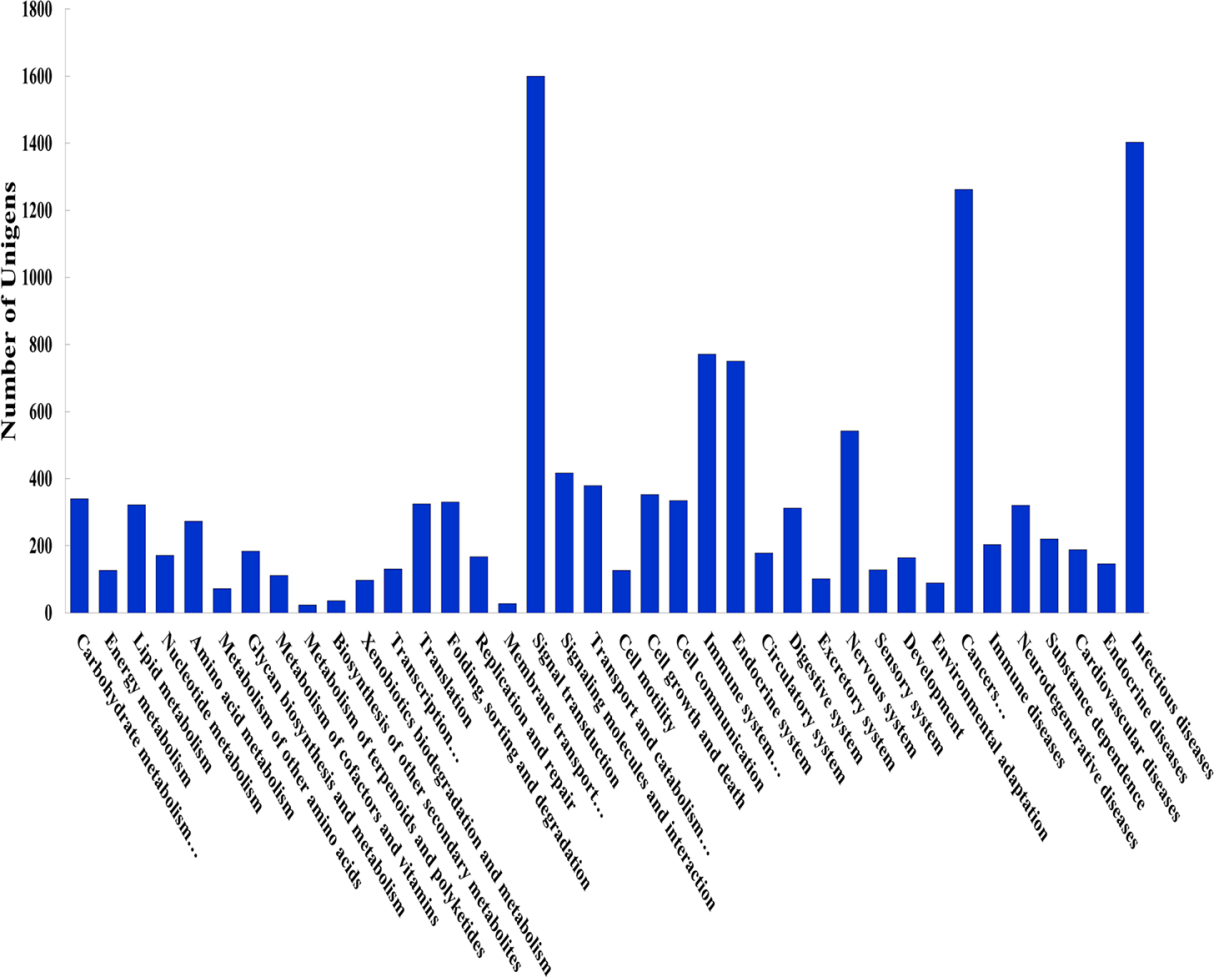
Eukaryotic clusters of orthologous group (KOG) classification. A total of 6088 sequences were clustered into 38 KOG categories.

KO is used to abbreviate KEGG (Kyoto Encyclopedia of Genes and Genomes) ontology; the KEGG database mainly provides data for metabolic pathways and annotations [18]. In the KEGG database, 6088 unigenes were first identified and categorized according to possible functions. All functions were divided into six clusters, including Metabolism, Genetic Information Processing, Environmental Information Processing, Cellular Processes, Organismal Systems and Diseases. The top pathway with highest representation of unigenes was “Diseases” (3744, 29.05%) (Fig 4). It should be that this was particularly evident in caged Lueyang black-bone chickens, which may explain the relatively weak immune or defense system found in caged poultry.

Among the 38 KOG categories, the largest group was the “Signal transduction” cluster (1600, 12.41%), followed by “Infectious diseases” (1403, 10.89%) and “Cancer” (1262, 9.79%); the smallest ones were “Metabolism of terpenoids” (24, 0.19%) and “Polyketides and Membrane transport” (28, 0.22%). IMP synthesis and degradation belong to nucleotide metabolism, and 172 unigenes were involved in the “Nucleotide metabolism” cluster (1.33%) in this study. The largest group was “Signal transduction”, corroborating other reports [19,20]. Therefore, the current findings further confirmed that “signal transduction” may be very important in the chicken skeletal muscle.

Using the KEGG database, we intuitively observed the location information of every expressed gene in metabolic pathways. In this work, a total of 6088 were divided into 753 KEGG pathways. At 60 days, the top three pathways were “PPAR signaling pathway”, “cancer pathway” and “AMPK signaling pathway”. At 120 days, “cancer pathway”, “PI3K-Akt” and “Muscle contraction signaling pathways” were the three most represented. The PPAR signaling pathway mainly regulates lipid metabolism, and involves PPARγ and PPARδ signal transduction. In young chickens, lipid metabolism is the most important in both caged and free-range animals. The above results suggested that PPAR signaling was most involved in all metabolic pathways, consistent with previous findings [21]. Currently, multiple studies have shown that many genes are associated with cancer occurrence and development, and involved in different signaling pathways. In particular, the associations of cancer with AMPK and PI3K-Akt signaling pathways have been demonstrated. AMPK and PI3K-Akt signaling pathways are involved in many cellular processes such as metabolism, inflammation, cell survival, cell movement and cancer. In this study, many cancer metabolisms related genes were detected in Lueyang black-bone chickens under both feeding patterns.

### IMP metabolism relevant genes and validation by qPCR

Previous reports found that 5-phosphate ribose pyrophosphate is a raw and processed material in IMP synthesis, which involves the purine metabolic pathway. Therefore, we particularly focused on the "purine metabolism" pathway in the "nucleotide metabolism group" in KEGG. In the nucleotide metabolism pathways, 172 important unigenes were identified, including 8 that were differentially expressed with a fold change cutoff of 2 in both feeding modes at 60 and 120 days, respectively (Table 5).

**Table 5:** Differentially expressed genes involved in IMP synthesis and degradation.

Ensembl ID	Gene Symbol	Gene Description	Fold Change	
			F 60d / C 60d	F 120d/ C 120d
ENSGALG12034	ADSL	Adenylosuccinate lyase	1.8328	0.98188
ENSGALG02082	AMPD1	AMP deaminase 1	5.2438	0.8298
ENSGALG26860	AMPD2	Adenosine monophosphate deaminase 2	1.4596	1.206
ENSGALG15983	GART	Phosphoribosyl-glycinamide formyl-transferase, phosphoribosyl-glycinamide	1.8396	1.969
ENSGALG03570	ATIC	5-aminoimidazole-4-carboxamideribonucleotide formyl transferase/IMP cyclo-hydrolase	1.071	1.203
ENSGALG05694	GARS	Glycyl-tRNA synthetase	1.683	0.896
ENSGALG08932	ENTPD8	Ectonucleoside triphosphate diphosphohydrolase 8	-	30.540
ENSGALG03816	NT5C1A	5'-nucleotidase, cytosolic IA	3.465	1.320

Note: F 60d, free-range group at 60 days; F120, free-range group at 120 days; C 60d, caged animals at 60 days; C 120d, caged chickens at 120 days; -, the gene doesn’t express in caged animals at 60 days.

Interestingly, 3 genes related to IMP metabolism were significantly different between the two culture modes (Table 5). In 60-day-old animals, AMPD1 (AMP deaminase 1), NT5C1A (5'-nucleotidase, cytosolic 1A) and ENTPD8 (ectonucleoside triphosphate diphosphohydrolase 8) were detected, while only ENTPD8 was found at 120-days. All IMP metabolism related genes were up-regulated in the free range group.

To further assess differentially expressed genes involved in the IMP metabolic pathway, the genes differentially expressed with a fold change cutoff of 2 were considered for evaluation in IMP metabolism pathways (Table 5). The results showed that 2 additional genes (GART and ADSL) were significantly up-regulated in the free-range group compared with caged animals. At 120 days, GART, GARS and ADSL in free-range chickens also showed higher levels than the values of caged chickens. In both 60 and 120 day-old chickens, IMPDH expression levels were lower in the free-range group compared with caged animals.

**Fig 5:**
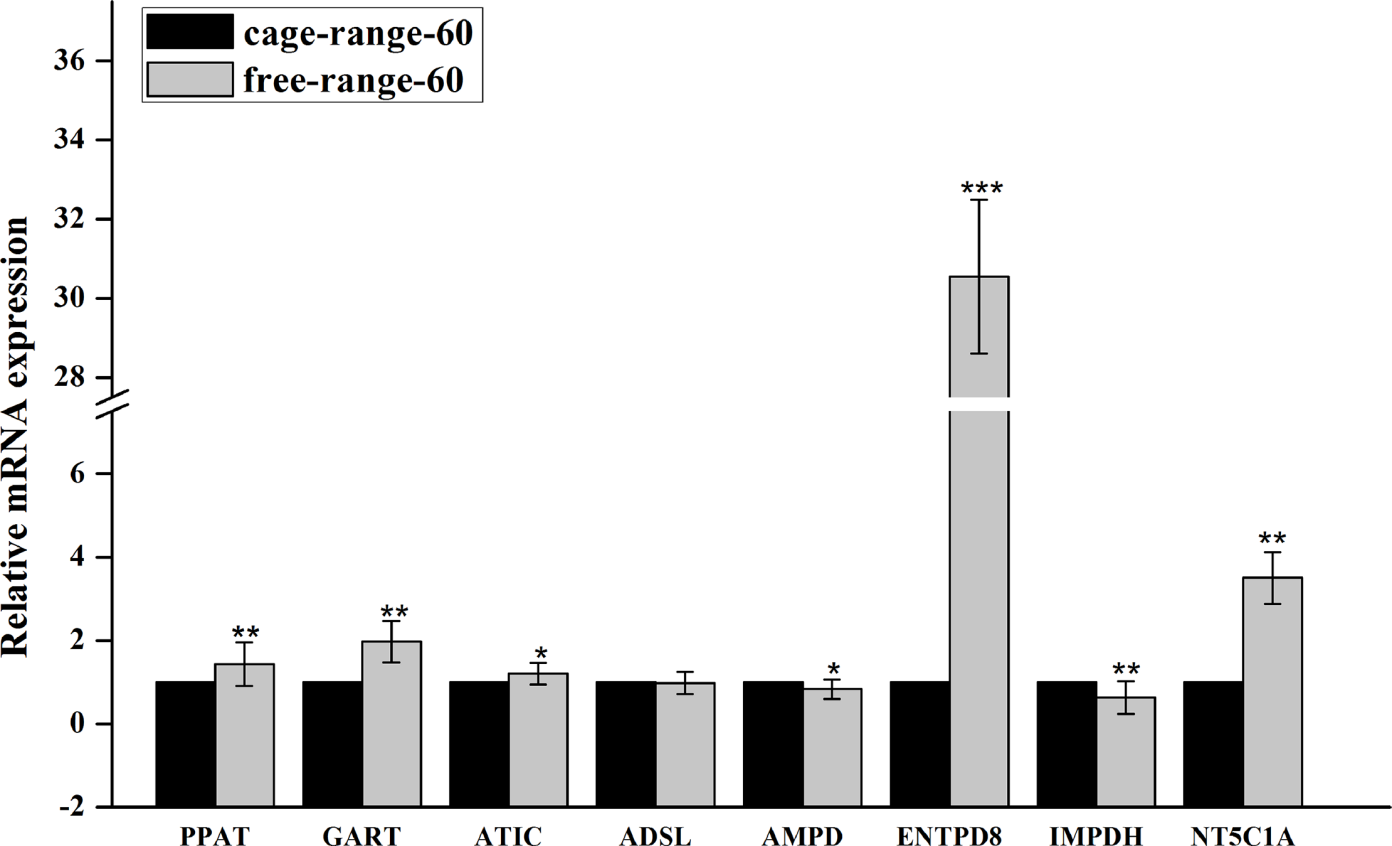
Expression levels of seven IMP-related genes validated by qPCR in thigh muscles from 60-day-old caged and free-range Chickens.

**Fig 6:**
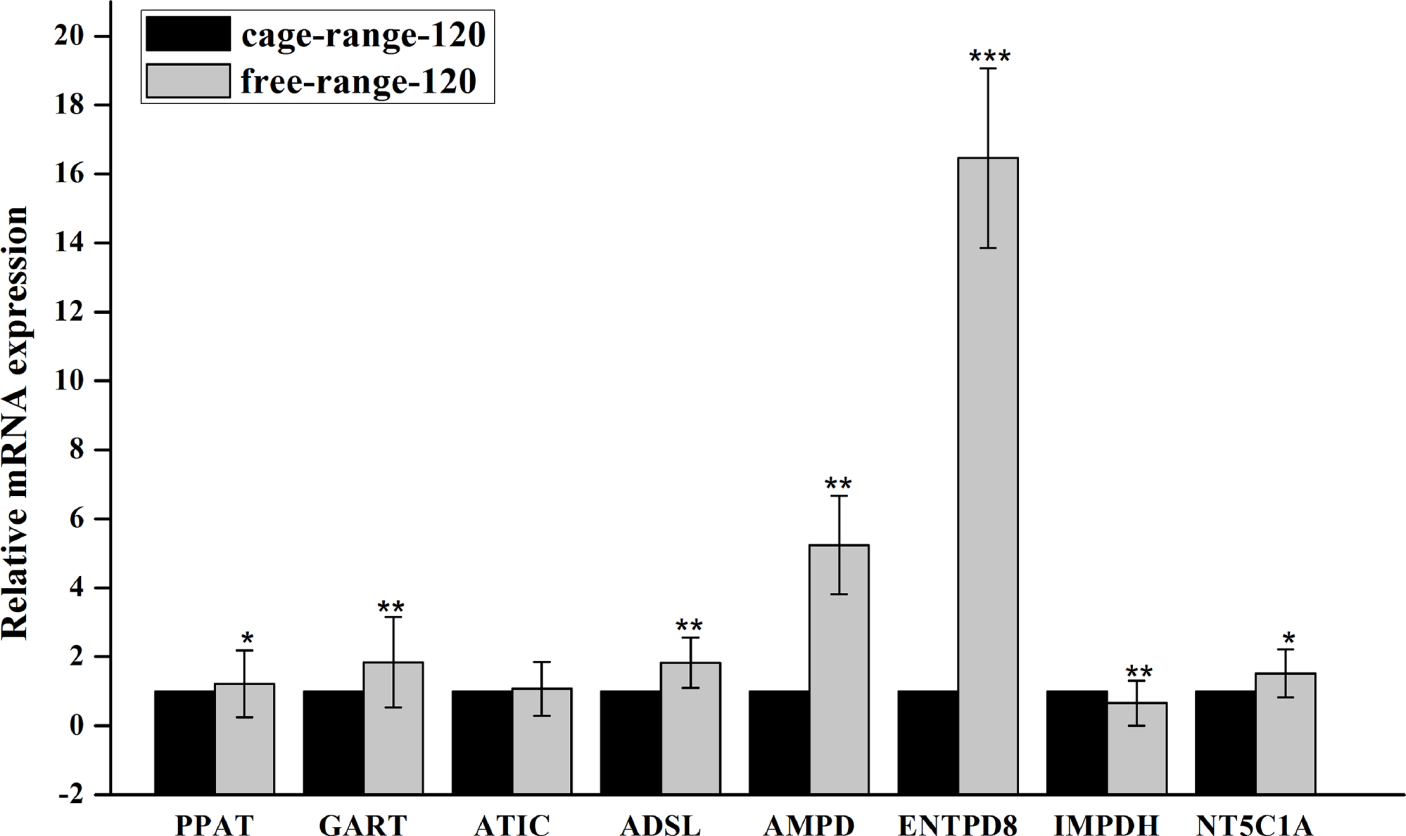
Expression of seven IMP-related genes validated by qPCR in thigh muscles from 120-day-old caged and free-range chickens.

Finally, to verify the reliability and validity of transcriptome data, qPCR was used to examine the expression levels of candidate genes in each group. As shown in Fig 5, at 60 days of age, PPAT, GART, ENTPD8, NT5C1A and ATIC were up regulated, but AMPD1 was down regulated in free-range chickens, and ADSL expression levels showed no differences between the two rearing modes, contradicting with transcriptome data. At 120 days of age, GART, ENTPD8, NT5C1A and ADSL expression levels were significantly increased in free-range animals, while a differential expression of PPAT and ATIC was not detected by qPCR (Fig 6). In both 60 and 120 day old animals, IMPDH expression levels were reduced in the free-range group. The above qPCR data were consistent with transcriptome data at some gene loci, further confirming that PPAT, GART, ENTPD8, AMPD1, ADSL and IMPDH expression levels were affected by the mode of chicken breeding at different growth stages.

## Discussion

It is well known that inosinic acid is a key factor that influences the fleshy flavor of meat, and an important indicator of meat flavor [22]. Therefore, many scientists pay close attention to inosinic acid synthesis and degradation in different livestock and poultry. The mechanisms of inosinic acid metabolism are complex, and a number of studies have revealed metabolic pathways and genes related to inosinic acid in meat [23]. The final amount of generated IMP is determined by three pathways, *de novo* synthesis, salvage pathway and IMP transformation [24].

There are metabolic steps and 8 key enzymes involved in *de novo* synthesis of IMP, including PPAT which is one of the key enzymes that catalyze the first step in *de novo* synthesis of this important molecule [25]. Glycamide nucleotide trans-methylase (GART) is an enzyme that catalyzes the fifth step of purine synthesis [26]. Inosine cyclase (ATIC) is a bifunctional enzyme that controls two enzymatic activities in the purine nucleotide metabolic pathway and plays a key regulatory role in the synthesis of inosinic acid [27]. Adenosine succinate lyase (ADSL) is another bifunctional enzyme that catalyzes the synthesis and cycling of purine nucleotides in IMP synthesis, as the key rate-limiting enzyme in the eighth step of purine synthesis. ADSL maintains the ATP/AMP ratio in the muscle tissue, and plays a major role in the final inosinic acid content of muscles [28,29]. Therefore, the ADSL gene has attracted attention in meat flavor research. In Lueyang black-bone chickens, ADSL, GART and ATIC showed higher expression levels in free-range animals than in caged counterparts at both 60 and 120 days of age. Taken together, these findings indicated that the free-range mode improves the expression levels of enzymes involved in IMP synthesis, thereby accelerating the *de novo* synthesis of IMP in chickens.

In the salvage pathway of IMP synthesis, hypoxanthine phosphoribosyl transferase mainly transforms hypoxanthine and 5-phosphate ribose pyrophosphate to hypoxanthine nucleotide. Studies reported that Entpd8 could catalyze the transformation of 5'-triphosphate inosine (ITP) into 5'-diphosphate inosine (IDP), and Entpd8 widely regulates many genes involved in IMP metabolism [30]. Therefore, the salvage pathway of IMP synthesis is affected by the complex regulatory network of Entpd8. In this study, Entpd8 expression was significantly increased in the free-range group, and the expression level of NTC5A1 also has similar phenomenon. NT5C1A plays an important role in nucleic acid and amino acid metabolism and is indirectly involved in the IMP metabolism pathway [31]. Combined with IMP content data, we speculate that the free-range mode may increase Entpd8 and NTC5A1 expression, subsequently enhancing IMP content in the chicken muscle by a synthesis compensation mechanism. Entpd8 and NTC5A1regulates IMP-related genes through the synthesis compensation pathway and thus affects the skeletal muscle via the salvage pathway.

The third related metabolic pathway is responsible for IMP transformation. *In vivo*, IMP is partially converted to ATP, providing a direct source of energy for various physiological activities. In addition, IMP could be converted to GMP by IMPDH. In the present study, IMPDH expression levels were significantly reduced in free-range animals, regardless of age. This indicated that free-range breeding could reduce IMP transformation by IMPDH regulation. On the other hand, ATP has a dynamic balance between synthesis and degradation. When the animals exercise, ATP consumption gradually increases in the muscle, and the degradation rate is greater than the synthesis one. This results in increased amounts of ADP, which is transformed into AMP by creatine kinase. Subsequently, AMP is transformed into IMP under the action of adenylate dehydrogenase. AMPD1, a member of the AMPD family, is a key enzyme in the metabolic pathways of ATP to AMP to IMP in the animal muscle[32]. Studies have reported that muscular IMP content gradually increases with chicken growth [33]. This study found that IMP content in the breast muscle showed an upward trend with increasing age in both feeding patterns; in addition, IMP levels in the thigh muscle showed the same trend in the free-range group. These results corroborated previous studies [34]. Nevertheless, an opposite trend was obtained in thigh muscle samples from the cage feeding group. This difference may be attributed to the amount of exercise of thigh muscles under distinct feeding modes. The motor function of muscles affects the dynamic balance of ATP, whose synthesis is reduced during exercise, with increased degradation. AMPD1 plays an important role in ATP catabolism. In free-range chickens, AMPD1 expression levels were increased at different ages, and significantly at 60 days, which is beneficial to promote AMP accumulation in the skeletal muscle. On the other hand, IMPDH activity in free-range chickens was significantly lower than that of caged counterparts at different ages. These findings indicated that the free-range mode could reduce IMP conversion and maintain a high level of IMP in the muscle by regulating IMPDH expression.

Through GO and KEGG pathway analyses, many differentially expressed genes associated with related metabolite pathways were detected, of which “nucleotide metabolism” and “purine metabolism” were further assessed. In the molecular function category, 5 significant genes were involved in nucleotide metabolism, with 3 related to IMP metabolism. Additional IMP metabolism related genes were identified in this study, although fold changes were somewhat below 2. Taken together, these findings provided strong evidence of a significant difference in IMP levels in chicken meat between free-range and caged animals. Poultry rearing patterns determine individual movement, inosinic acid deposition and body weight, among other parameters. The main issue is how to design a good method to achieve an optimal balance between meat quality and growth rate. Therefore, further investigation is required to unveil the regulatory relationships within these genes and the key factors of feeding patterns.

## Conclusion

Chickens have a wider range of intake, larger amounts of exercise and much more energy consumption in the free range mode compared to cage breeding. The former feeding mode is healthier and meets the needs of animal welfare, and facilitates inosinic acid deposition in the muscle. This increases muscle freshness and improves meat flavor. The present study is an original report of the transcriptome analysis of muscle samples from free-range and caged chickens. The obtained data described and revealed a set of differentially expressed genes in chickens under different feeding modes that are potentially related to IMP synthesis and degradation.

Collectively, these findings provide a valuable theoretical basis for designing an efficient and healthy poultry feeding mode, as well as a foundation for further studies assessing IMP accumulation in chicken meat.
